# Contamination of Dental Surgical Masks by Aerosols Generated During Different Dental Treatments

**DOI:** 10.3290/j.ohpd.b5866891

**Published:** 2024-12-03

**Authors:** Alexandra Stähli, Rui Fang Nhan, Janika Michelle Schäfer, Jean-Claude Imber, Andrea Roccuzzo, Anton Sculean, Martin Schimmel, Christian Tennert, Sigrun Eick

**Affiliations:** a Alexandra Stähli Assistant Professor, MAS Staff Member, Department of Periodontology, School of Dental Medicine, University of Bern, Bern, Switzerland. Idea and experimental design, wrote the manuscript, read and contributed to the manuscript.; b Rui Fang Nha Doctoral Student, Department of Periodontology, School of Dental Medicine, University of Bern, Bern, Switzerland. Prepared the masks, sterile filter papers, helped with data collection, read and contributed to the manuscript, *contributed equally to the manuscript and share second authorship.; c Janika Michelle Schäfer * Doctoral Student, Department of Periodontology, School of Dental Medicine, University of Bern, Bern, Switzerland. Prepared the masks, sterile filter papers, helped with data collection, read and contributed to the manuscript, *contributed equally to the manuscript and share second authorship.; d Jean-Claude Imber Periodontologist, MAS Staff Member, Department of Periodontology, School of Dental Medicine, University of Bern, Bern, Switzerland. Wore the masks during treatment, read and contributed to the manuscript.; e Andrea Roccuzzo Periodontologist, MAS Staff Member, Department of Periodontology, School of Dental Medicine, University of Bern, Bern, Switzerland. Wore the masks during treatment, read and contributed to the manuscript.; f Anton Sculean Full Professor and Chair, Department of Periodontology, School of Dental Medicine, University of Bern, Bern, Switzerland. Idea and experimental design, wore the masks during treatment, read and contributed to the manuscript.; g Martin Schimmel Full Professor and Chair, Department of Reconstructive Dentistry and Gerodontology, School of Dental Medicine, University of Bern, Switzerland. Wore the masks during treatment, read and contributed to the manuscript.; h Christian Tennert * Professor, MAS Staff Member, Department of Restorative, Preventive and Pediatric Dentistry, School of Dental Medicine, University of Bern, Switzerland. Idea and experimental design, wore the masks during treatment, read and contributed to the manuscript.; i Sigrun Eick * Professor and Head, Oral Microbiology Laboratory Department of Periodontology, School of Dental Medicine, University of Bern, Bern, Switzerland. Idea and experimental design, conducted the statistical analysis, wrote the manuscript, read and contributed to the manuscript,

**Keywords:** aerosols, dental care, dental care team, masks

## Abstract

**Purpose:**

The COVID-19 pandemic raised the question about the extent of microbial exposure encountered by dentists during dental therapy. The purpose of this study was to quantify microbial counts on surgical masks related to duration and type of dental therapy, as well as patient oral health variables.

**Materials and Methods:**

Sterile filter papers were fixed on surgical masks used during routine daily dental therapy. Thereafter, the filter papers were pressed onto blood agar plates for 1 min, before the agar plates were incubated with 10% CO2. After 48 h, the colony forming units (CFU) were counted and microorganisms were identified. The dependence of the CFU counts on treatment and patient-related variables was analysed using linear regression.

**Results:**

Filter papers obtained from 322 dental treatments (429 masks) were included in the final analysis. On average, 5.41 ± 9.94 CFUs were counted. While mostly oral bacteria were detected, Staphylococcus aureus was also identified on 16 masks. Linear regression, incorporating patient-related and treatment characteristics through step-wise inclusion, revealed statistical significance (p < 0.001) only with the variable “assistance during therapy”. The type of dental treatment exhibited a trend, with fewer CFUs observed in caries treatment compared to periodontal or prosthodontic therapy. Furthermore, after analysing filter papers from masks used by dental assistants in 107 dental treatments, fewer CFUs were found on the masks compared to those used by dentists (p < 0.001).

**Conclusion:**

The mean number of CFUs observed consistently remained low, highlighting the efficacy of the implemented hygiene measures. Consequently, it is clinically recommended to support dental treatment with precise suction of the generated aerosols.

Aerosols are defined as minute solid or liquid particles suspended in air. Dental health care professionals face a significant risk of exposure to aerosols generated during various dental procedures that involve water cooling or rotating instruments, such as the use of low- or high-speed handpieces, ultrasonic scalers, air-polishing devices, or air and water syringes. In addition to microorganisms, these aerosols contain a variety of particles, including calcium, phosphorus, carbon, aluminum, iron, nickel, or tungsten, which can be inhaled. When these particles have a diameter less than 10 µm, they can enter the nose or pharynx; if they have aerodynamic diameters < 5 µm, they can even reach deeper regions of the lungs.^23^ However, most aerosols generated by dental procedures contain droplets larger than 50 µm.^23^

The outbreak of severe acute respiratory syndrome coronavirus 2 (SARS-CoV-2) in 2019 and its subsequent global spread have brought serious health concerns to the forefront, particularly regarding the transmission of pathogens. SARS-CoV-2 primarily spreads through airborne transmission via respiratory droplets and aerosols.^14^ While breathing, speaking, coughing, or sneezing, aerosols are being generated, which are able to carry pathogens. During active phases of infection, a person can release a large number of these airborne microorganisms. The SARS-CoV-2 spike protein specifically recognises and invades cells via the angiotensin-converting enzyme (ACE) 2, which is very abundant in epithelial cells of the tongue, oral mucosa, gingiva, and salivary gland ducts, making the oral cavity a major route for the spread of the infection.^17,30^

In addition to viruses, aerosols can also transmit bacteria from patients to dental professionals. In this respect, the colonisation of potential pathogens in the oropharynx of dental professionals has been reported. A study on nasal carriage of Staphylococcus aureus revealed a higher percentage of positive results for individuals with direct contact to the patients’ oral cavities compared to those without.^16^ In regions with a high prevalence of MRSA, 6.4% of dental students with clinical experience were colonised with MRSA, whereas MRSA was not detected in the noses of dental students without such experience.^2^

To prevent the transmission of pathogens in dental practices, an infection control regimen is a fundamental standard, as in any dental facility. In addition to rinsing with an antiseptic solution before treatment, surface disinfection, autoclaving of instruments, and other protocols, protective equipment, including gloves, masks, eye protection, protective clothing, and surgical head caps, is used.^28^ The generation of aerosols, environmental contamination, and the effectiveness of protective equipment depend not only on the specific treatments employed,^3^ but also on the use of a high-volume evacuator (HVE).^24^ While most routine dental procedures profit from assistance and efficient HVE, dental hygiene treatments are typically conducted by a single operator, often in infected oral environments, without assistance. Furthermore, due to the current shortage of skilled workers in certain European countries, a two-handed treatment protocol has often become necessary.

To date, there is limited knowledge regarding mask contamination following dental treatments, particularly with regard to differences between single- and two-operator procedures. Hence, the aim of this study was to assess and compare the microbial counts on masks across various routine dental treatment modalities. To accomplish this, we conducted an evaluation of 429 masks obtained from three different dental clinics within the University of Bern, Switzerland.

## MATERIALS AND METHODS

Masks worn by dental health care professionals (dentists, dental hygienists, dental assistants) were analysed. Three different departments of the School of Dental Medicine, University of Bern, Bern, Switzerland (Department of Periodontology, Department of Restorative, Preventive and Pediatric Dentistry; the Department of Reconstructive Dentistry; and Gerodontology) participated in this study. All routine treatment procedures performed daily were included. In order to include masks in the analysis, patients undergoing treatment had to be over 18 years of age and willing to participate in this research.

Ethical approval and informed consent: All procedures performed in this study were in accordance with the ethical standards of the institutional ethics committee and with the 1964 Helsinki Declaration and its later amendments or comparable ethical standards. The study was approved by the ethics authorities of the Canton of Berne (Berne, Switzerland; ID 2020–02706). Written informed consent was given by all patients who received treatment with the hygiene masks that were analysed in this study.

### Patients and Dental Treatment

Patient characteristics included age, gender, systemic conditions such as diabetes mellitus, obesity (BMI > 30), chronic obstructive pulmonary disease (COPD), rheumatoid arthritis, immunomodulatory medication, and smoking status. Among clinical oral variables, data included the number of teeth, utilisation of removable dental prostheses, periodontal screening and recording index (PSR), oral hygiene as measured using the O’Leary plaque record,^22^ assessed at 4 sites per tooth (with a reference for good oral hygiene, distinguishing it from suboptimal oral hygiene when the plaque index was > 0.25), decayed-missing-filled surface index (DMFS), and the number of decayed surfaces.

The treatment modality, including conservative, periodontal, surgical, and endodontic procedures, was carefully documented. Additionally, whether or not a rubber-dam was used and whether or not an assistant was present (i.e., four-handed procedure) was recorded. The duration of treatment and aerosol generation completed the list of data generated by the treatment provided.

### Mask Preparation

Two 5x5-cm filter papers (Schleicher & Schüll; Feldbach, Switzerland) were clipped onto the masks. Thereafter, each mask was placed in an envelope (Wipak medical, Steriking; Bomlitz, Germany), sterilised and consequently worn by the operators (i.e., dentist, dental assistant, and dental hygienist) for the whole duration of the intervention. After treatment, the filter papers were removed with sterile tweezers, immediately placed onto agar plates and transported to the laboratory.

### Microbiological Analysis

In the laboratory, one filter paper was stored at -80°C (designated for detection of influenza and SARS-CoV-2 virus). Using tweezers, the second filter paper was pressed onto agar plates containing 5% of sheep blood (contact) for 10 min and thereafter discarded. After incubation at 37°C with 10% CO2 for 48 h, bacteria and fungi were counted (colony forming units [CFU]) on the agar plates. According to colony morphology and other routine methods (Gram staining, hyaluronidase for Staphylococcus aureus), the microorganisms were grouped into oral microorganisms, other commensals, potential pathogenic strains, bacilli and fungi. All potential pathogenic strains or strains with a potential multi-drug resistance were isolated, subcultivated, identified, and stored at -20°C (secondary outcome). A later analysis determined selected antimicrobial resistance. Cefoxitin disks were used to detect methicillin resistance in S. aureus strains according to the EUCAST guidelines.

### Statistical Analysis

Preliminary data identified an average count difference of 25% on dental masks between treatment with and without assistance. Therefore, the power analysis for the present study assumed this difference with the same standard deviation of 20%. Enrolling a total of 50 volunteers per group should yield a statistical power of 80 at an alpha level of 5%.

As secondary outcomes, patient- and treatment-related variables as indicated above. Additionally, microbial profiles were analysed.

Statistical analysis included descriptive statistics and the use of non-parametric tests (Mann-Whitney test, and Wilcoxon test for dependent groups). In addition, multiple linear regression with stepwise inclusion was performed to assess the influence of patient and treatment characteristics on the microbiological outcome. The level of significance was set at p = 0.05. Software SPSS 29.0 (IBM SPSS Statistics; Chicago, IL, USA) was used.

## RESULTS

The analysis was conducted on masks used in 322 treatments (219 from the Department of Periodontology, 85 from the Department of Restorative, Preventive and Pediatric Dentistry, and 18 from the Department of Reconstructive Dentistry). All treatments were performed between March and September 2021. This comprised a total of 429 mask samples. Additionally, 107 masks worn by dental assistants were included in a subgroup analysis.

Data are available from the corresponding author upon request.

### Treated Patients and Treatments Provided

Patient characteristics are summarised in Table 1. Of all participants, 133 (45.5%) were men and 159 (54.5%) women, 10 (3.1 %) had diabetes mellitus, 3 (0.9%) had a BMI of 30 or more, and 4 (1.2%) suffered from diseases such as chronic obstructive pulmonary disease or rheumatoid arthritis. The number of recent COVID infections was low and no single case of seasonal influenza was recorded. Therefore, it was decided not to further analyse the filter paper samples on masks for the presence of these viruses.

**Table 1:** Epidemiologic and anamnestic data of the treated patients

Variable	Patients’ data available (n)	Results
Gender	292	Male 133 (45.5%), female 159 (54.5%)
Age (years)	266	Mean ± SD: 57.1 ± 15.2; range: 18-93
Diabetes mellitus	291	10 (3.1%)
BMI ≥ 30	292	3 (0.9%
Other systemic diseases (e.g., COPD, RA)	292	4 (1.2%)
Intake of antibiotics within the last month	292	21 (6.5%)
Intake of drugs affecting the immune system	292	20 (6.8%)
COVID infection within the last month	289	4 (1.4%)
Smoking	288	No 194 (67.4%); yes 63 (21.9%); former 31 (10.8%)


Patients had an average of 25 natural teeth in their oral cavity. Less than 10% of the patients wore dentures. Half of the patients had good oral hygiene (i.e., plaque index up to 0.25), the other half did not. There was a high variability related to treated and untreated carious lesions (Table 2).

**Table 2:** Oral health clinical data of the patients

Variable	Patients’ data available (n)	Results
Number of teeth	275	§ 24.3 ± 4.9; range: 0–32
Wearing of dentures	287	yes: 23 (8%), no: 264 (92%)
Oral hygiene	278	good: 157 (56.5%), not optimal: 121 (43.5%)
Mean PSR	280	§ 2.40 ± 1.11; range: 0–4
DMFS	273	§ 13.7 ± 8.7; range: 0–54
Decayed surfaces	279	§ 0.7 ± 2.1; range 0–18
§Mean ± SD

Treatment data were available for 289 subjects. Periodontal therapy was provided in 176 (60.9%), endodontic therapy in 16 (5.5%), surgery in 26 (9%), and conservative treatment (filling/prosthetic) in 71 (24.6%) patients. Of the 292 cases analysed, 224 (69.6%) were treated with assistance (four-hand) and 68 (23.3%) were treated by dental hygienists. Rubber-dam was used in 43 (14.7%) of the 292 treatments.

### Bacteria/Fungi on the Masks of the Dentist and Dental Hygienist

**Fig 1:**
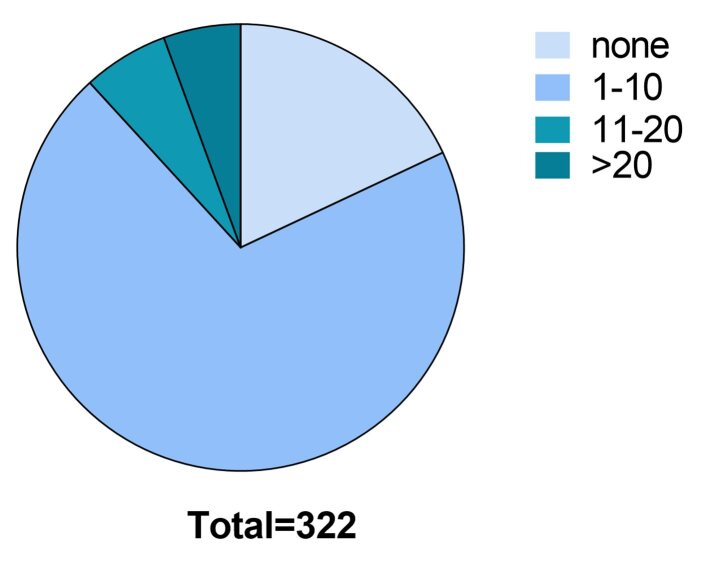
Counts of microorganisms (CFU of bacteria and fungi) on 322 dental hygiene masks.

When analysing the 322 masks of the main treatment providers, a mean of 5.41 ± 9.94 CFU were counted on the agar plates exposed to the filter papers of the masks. In 58 (18%) of the samples, no CFU was found. In the majority of cases (226, 70.2%), up to 10 CFU were detected on the filter papers, but in 18 (5.6%) samples more than 20 CFU (up to 75 CFU) were detected (Fig 1).

Most of the microorganisms identified on the masks were oral bacteria or commensals (Table 3). On 17 masks, S. aureus was identified. Resistance testing against cefoxitin showed that MRSA was not present among the S. aureus strains. Additionally, when rubber-dam was used, no S. aureus was detected. Apart from S. aureus, only one potential multidrug-resistant bacterium, Pseudomonas aeruginosa, was identified. This bacterium was found as a single CFU on a mask following treatment of a patient with periodontitis. Importantly, this strain was susceptible to anti-P. aeruginosa antibiotics (piperacillin, ceftazidime, ciprofloxacin).

**Table 3:** Identified bacteria/fungi on 322 masks

Species group	N (%)
None	58 (18.0%)
Oral bacteria	204 (63.4%)
Commensals	164 (50.9%)
Staphylococcus aureus	17 (5.3%)
Aerobic gram-negative rods	1 (0.3%)
Bacilli	10 (3.1%)
Mould (fungi)	5 (1.6%)
In positive samples, often more than one species group was present.

### Colony Forming Units on Masks in Relation To Patients’ Anamnestic and Clinical Data and Treatment Procedures

CFU counts were related to patients’ data and different treatment aspects. Regression analysis, employing multiple linear regression with stepwise inclusion, yielded two models. In model 1 (p < 0.001) only the variable “working with assistance” (four-hand) was included. In model 2 (p = 0.022), aside from “working with assistance”, variables such as the number of teeth, wearing of dentures, mean PSR, oral hygiene, and DMFS were included. However, only “working with assistance” was statistically significant (p < 0.001). This implies that all patient-related variables, whether systemic or oral health-related, statistically significantly influenced the CFU counts on masks in the multivariate models.

**Fig 2:**
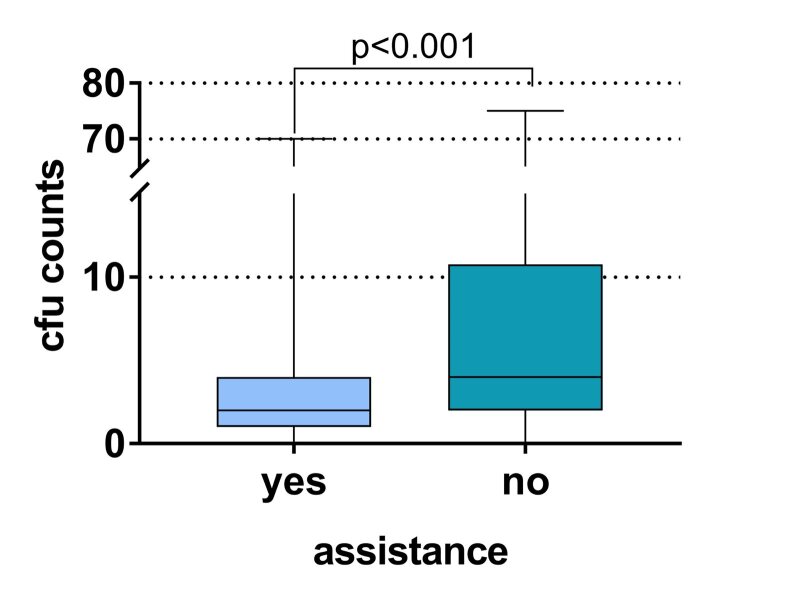
Counts of microorganisms (CFU of bacteria and fungi) on 322 dental hygiene masks related to provided treatment with (four-handed) and without assistance. Data are presented as medians with lower and upper quartile. Whiskers represent the maximum score.

A comparison of CFUs using the Mann-Whitney test confirms the finding, revealing higher CFU counts when working without assistance compared to with assistance (Fig 2). The mean numbers of CFU were 3.97 ± 7.79 with and 9.78 ± 14.51 without assistance (median: with assistance 2 CFU, without assistance 4 CFU).

In addition, the type of treatment provided did not show a statistically significant influence. There seemed to be a tendency to fewer CFU when conservative treatments (e.g., caries/endodontic treatment; in regression analysis as a combined variable) were performed. Subgrouping the evaluated treatments, a mean CFU count of 5.72 ± 10.07 was found for periodontal treatment, 4.05 ± 8.82 for caries treatment, 5.00 ± 8.45 for endodontic treatment, 5.88 ± 11.04 for prosthetic treatment, and 6.00 ± 13.78 for surgeries. In regression analysis, the use of rubber-dam made no difference: comparing treatments with and without, there were fewer CFU when rubber-dam was used (mean 2.58 ± 3.21 CFU) than when it was not (mean 5.80 ± 10.75 CFU; p = 0.026).

### Colony Forming Units on Masks of Dental Assistants 

In addition, 107 masks worn by dental assistants from the Department of Periodontology were analysed. Comparing these masks with those of the corresponding dentists revealed more numerous CFU on the dentists’ masks (2.95 ± 3.39) compared to dental assistants’ masks (1.28 ± 2.16; p < 0.001). Notably, nearly 50% of the filter papers from dental assistants’ masks tested negative, whereas about 25% of the dentists’ masks showed the same result. Consequently, the percentage of identified species groups was higher in the dentist group (Table 4).

**Table 4:** Identified bacteria/fungi on 107 masks of dental assistants and on 107 masks of the respective dentists

	Dental assistant (n = 107)	Dentist (n = 107)
None	53 (49.5%)	26 (24.3%)
Oral bacteria	35 (32.7%)	65 (60.7%)
Commensals	30 (28.0%)	43 (40.2%)
Staphylococcus aureus	1 (0.9%)	2 (1.9%)
Aerobic gram-negative rods	0	1 (0.9%)
Bacilli	1 (0.9%)	2 (1.9%)
Mould (fungi)	1 (0.9%)	2 (1.9%


## DISCUSSION 

In this study, we analysed the contamination of surgical masks, which serve as a protective shield for treatment providers during routine dental treatments. To mitigate the risk of exposure, face masks provide a physical barrier against particles that are 1 µm or larger in diameter. It has been demonstrated that masks are highly effective, reducing exposure by 86-92%.^5^ In addition to surgical masks, there are different levels of filtering-facepiece (FFP) protection available, ranging from FFP1 to FFP3. On average, FFP masks offer protection factors 11.5 to 15.9 times greater than surgical masks.^15^ Among FFP masks, FFP3 masks present the highest level of protection, reducing exposure to solid and liquid aerosols 20-fold compared with not wearing a mask.^4^ When comparing the effectiveness of surgical masks and N95 respirators (FFP2) in protecting healthcare workers against influenza, the use of surgical masks yielded infection rates similar to those of N95 respirators.^18^ Regarding SARS-CoV-2, both the surgical and N95 respirators reduce the risk of infection; however, N95 respirators might be more effective.^6^

This research was inspired by the discussion around virus transmission via aerosols in the context of the SARS-Cov2 pandemic. The objective of the present in-vitro study was to investigate whether different treatment modalities, treatment duration, or patient factors have an impact on surgical mask contamination, as represented by bacterial/fungal counts.

Our main finding was that irrespective of treatment duration and patient characteristics, microbial contamination of hygiene masks was low. The CFU numbers on masks were noticeably lower than those reported for masks used in hospitals, where 100 CFU/ml/piece were counted.^19^ This might be due to the strict infection control regimen established in our School of Dental Medicine. Our surgical masks derived from dental treatments that were mostly performed in rooms with open windows. Furthermore, masks were sterilised before use and exchanged after each patient, and the patients performed a pre-treatment oral rinsing with 1% hydrogen peroxide solution for 60 s, according to the recommendations of the Swiss Dental Society established during the first months of the SARS-CoV-2 pandemic. Gund et al^12^ reported that rinsing the oral cavity with an antiseptic, such as chlorhexidine, statistically significantly reduced the microbial load vs water rinsing alone. However, others did not observe a decrease in salivary viral load among SARS-CoV-2 positive patients when they rinsed with 0.005% cetylpyridinium chloride-0.05% chlorhexidine (CPC) mouthwash compared to rinsing with sterile water.^9^

It is important to mention that during pandemic, it was mandatory to use high-volume evacuation (HVE) in our School of Dental Medicine. The very low numbers of CFU may be due to its effectiveness. At the dentist’s and dental assistant’s nose, the use of HVE reduced the CFU counts by 83% and 81.5%, respectively, which was further decreased by 5% when the patients rinsed their mouths with an antiseptic for 1 minute prior to treatment.^8^ We have focused on surgical masks and on dental health care professionals in very close proximity to the patients’ mouths, but HVE also prevents the spread of potential infectious agents in a wider area.^10^

The primary aim of the study was to compare CFU counts on masks related to the treatment performed with and without assistance. Although the CFU counts were generally low, a higher microbial load was found on masks when treatment was performed without assistance. However, it must be mentioned that the dental hygienists use HVE, but HVE is either applied intermittently or it might not be exactly placed. To the authors’ best knowledge, comparing data with other studies is not possible. Nevertheless, the data presented would recommend working with an assistant (i.e., four-handed) whenever possible.

In this study, mostly oral bacteria were found on the masks; Staphylococcus aureus and molds were rarely detected, while other authors^19^ identified the predominant species to be Staphylococcus spp and Aspergillus spp. In an Iranian hospital during the early months of 2022, mostly Staphylococcus spp were detected, followed by Acinetobacter and Pseudomonas spp, while Klebsiella and Enterococcus spp were rarely found.^29^ Others reported that 240 masks collected from 130 dental healthcare professionals predominantly harbored staphylococci at a rate of 26.4%, Pseudomonas spp 17.8%, and streptococci in 15.5% of the masks.^25^ Aerobic gram-negative species were found on only one mask. This raises the question of whether the high percentages of gram-negative bacteria, staphylococci, and molds reported by others are from the patient’s oral cavity or environmental contaminants.

Although this study was conducted during a pandemic, the search for viruses was ultimately omitted, as at the time of the study, the number of influenza and SARS-CoV-2 infection was very low in the population. This might be a limitation of the study. Viral load was assessed on masks by Chughtai et al,^7^ who examined masks from 12 doctors and nurses working in infectious diseases, respiratory/chest wards and intensive care units in Sydney and from 158 doctors and nurses working in respiratory wards and fever clinics in pre-pandemic Beijing from December 2017 to January 2018. Of the 36 Sydney samples, only three tested positive for human enterovirus. In the samples of 158 masks from Beijing, virus positivity was 10.1% with adenovirus being most commonly found, followed by RSV and influenza virus.^7^ Virus positivity rates were higher, but not statistically significant, on mask from participants who worked with high-risk patients or performed aerosol-generating procedures.^7^

Surprisingly, while a high rate of healthcare workers contracted SARS-CoV-2 through nosocomial transmission, dental healthcare workers remained nearly unaffected.^26^ A national cohort study conducted in Israel revealed a notably low cumulative transmission rate of SARS-CoV-2 during dental treatments.^20^ Out of 962 instances in which dental staff were exposed to 508 patients who had tested positive for SARS-CoV-2, only seven infections (0.7%) occurred between May and September 2020. Similarly, the transmission rate from COVID-positive dental healthcare professionals to patients was also low, with a rate of 0.6%.^20^ It can be assumed that in Israel also a high standard of infection protective measures in dentistry exists.

Of course, the inner side of the masks would be a crucial site in terms of effective infection transmission. However, standardisation with operators disinfecting their face skin would have been more challenging and considered a potential source of errors. Obviously, higher contamination was found on the outer side of masks.^19,25^ Among the 230 surgical masks collected from wards of a government hospital in Bangkok, mean CFU counts were 166 ± 199 ml/piece on the outer sides and 47 ± 56/ml/piece on the inner sides. The counts in the air samples correlated with the bacterial load on the outer sides. The fact that CFU counts on the masks from the Bangkok hospital are conspicuously higher than in our study might be due to different local conditions and sampling conditions. In the Bangkok study, masks derived from staff working in intensive care units, emergency and operating rooms, and in an outpatient department of the hospital, while our masks derived from dental treatments that were mostly performed in rooms with open windows. Furthermore, as indicated previously, a pre-treatment rinsing of the patient’s oral cavity was performed. Other authors who also assessed masks from dental healthcare professionals found outside/inside mask areas with 180 ± 110 CFU/ml/piece and 48 ± 26 CFU/ml/piece, respectively.^25^

A few reports compare contamination of the orofacial areas of dentists with dental assistants. In part there was no clear difference,^8^ but a study focusing on blood contamination after oral surgeries found 2.5-fold higher positive results for blood spatters in operators than in assistants,^1^ which might be in line with our results.

Treatment with rubber-dam showed a trend toward lower CFU counts on masks, a well-established finding. In the 1980s, Samaranayake et al^27^ demonstrated the efficacy of rubber-dam usage in reducing bacterial contamination in the environment, reporting as much as a 70% reduction in airborne bacterial particles.

It might seem plausible that patients harbouring a great amount of biofilm and inflamed tissues might be more likely to produce a bacteria-infested aerosol. However, no correlation between periodontal screening index or inflammatory periodontal state and the level of mask contamination was found in our study. Similarly, masks worn by healthcare workers with varying degrees of patient exposure were assessed, revealing no statistically significant differences among them.^21^ Furthermore, no difference was observed between healthcare workers who had contact with patients infected with multidrug-resistant organisms and those who did not.^21^

The absence of a clear association between microbial counts on surgical masks and the administered therapy is in line with a different study,^11^ which examined hygiene masks and gloves worn during various treatment modalities, including carious cavity preparation, tooth substance preparation, trepanation, root canal treatment, as well as supra- and subgingival periodontal ultrasonic instrumentation. Similarly, that study failed to identify statistically significant differences in bacterial contamination levels.^11^

The present study has some limitations. Firstly, the study was conducted during March and November 2021 as the peak of the pandemic was declining and with more spring to summer months where viral infections are lower than in winter months. Secondly, the inside surfaces of masks were not evaluated, which would be considered the ultimate endpoint when evaluating the risk for infection. However, we did not seek to evaluate the effectiveness of dental masks but rather the mask contamination resulting from one of the most aerosol-producing fields in medicine where oral transmission of diseases might seem most obvious. Interestingly, the present results indicate that bacterial loads during dental treatments are generally low. Using rubber-dam and performing 4-handed treatment further lowered CFU counts statistically significantly.

This study did not assess differences in mask contamination between experienced and inexperienced operators, such as graduate students. Others have assessed the contamination of dental masks in second-year students performing a variety of treatments, including periodontal and endodontic treatments, taking an average of 120 minutes per treatment.^13^ However, they did not report CFU counts on the masks; neither did they compare mask contamination with that of more experienced operators. We can only assume that the masks may have a different bacterial profile due to longer treatment times with multiple interruptions, where students are more likely to touch their masks with bare fingers. This, rather than the duration of treatment, could possibly result in higher contamination (more CFU). However, this needs to be investigated further.

The very low mean number of CFU on masks may support the implementation of effective infection control in dental practices to prevent transmission of pathogens. Assistance during dental treatment, allowing a tightly focused suction of the generated aerosols, is recommended.

## ACKNOWLEDGEMENTS

The laboratory work of Prashanthnj Sivapatham and Ivan Abegglen (Department of Periodontology, Laboratory of Oral Microbiology, University of Bern, Switzerland) is greatly appreciated. We are grateful to the dental hygienists Barbara Blaser, Franziska Hofmann, Barbara Iseli, Eva Läderach, and Eva Lütge for their enthusiastic efforts. Thanks also go to Christoph Ramseier for sound statistical advice and discussions. Alexandra Stähli is supported by a 3-year grant from the Geistlich-Stucki Foundation. This study was entirely funded by the Department of Periodontology of the University of Bern.

## REFERENCES 
